# The Monocytes That Repopulate in Mice After Cyclophosphamide Treatment Acquire a Neutrophil Precursor Gene Signature and Immunosuppressive Activity

**DOI:** 10.3389/fimmu.2020.594540

**Published:** 2021-01-25

**Authors:** Zhi-Chun Ding, Nada S. Aboelella, Locke Bryan, Huidong Shi, Gang Zhou

**Affiliations:** ^1^Georgia Cancer Center, Medical College of Georgia, Augusta University, Augusta, GA, United States; ^2^Department of Biochemistry and Molecular Biology, Medical College of Georgia, Augusta University, Augusta, GA, United States; ^3^Department of Medicine, Medical College of Georgia, Augusta University, Augusta, GA, United States

**Keywords:** chemotherapy, myeloid cell, monocyte, neutrophil, immunosuppression

## Abstract

Cyclophosphamide (CTX) is a major component of the chemotherapy conditioning regimens used in the clinic to prepare cancer patients for hematopoietic stem cell transplantation or adoptive T cell therapy. Previous studies have shown that CTX given at nonmyeloablative doses in mice and patients leads to expansion of myeloid cells within which the monocytic subset exhibits immunosuppressive activity. However, the ontogeny and gene expression signature of these CTX-induced monocytes are not well-defined. Here, we report that the expansion of myeloid cells is a default process intrinsic to hematopoietic recovery after chemotherapy. During this process, the monocytes repopulated in mice acquire immunosuppressive activity, which can persist long after cessation of chemotherapy. Moreover, monocytes acquire a gene signature characteristic of neutrophil precursors, marked by increased proliferative capability and elevated expressions of multiple primary and secondary granules. We provide evidence that CTX-induced myeloid cell expansion is regulated by DNA methyltransferase 1 (Dnmt1) and dependent on chemotherapy-induced microbial translocation. These findings help advance our understanding of the differentiation, heterogeneity, and function of myeloid cells repopulating after chemotherapy.

## Introduction

Chemotherapy is one of the major treatment modalities for patients with cancer. It is increasingly clear that elements of the tumor microenvironment (TME), especially cells of the myeloid lineage, critically influence host response to chemotherapy (1–4). There is ample evidence that tumor-derived factors, such as IL-6, TNFα, and PGE2, induce aberrantly differentiated myeloid cells, such as myeloid-derived suppressor cells (MDSCs) and tumor-associated macrophages (TAMs), which mediate immune suppression and promote tumor cell survival, metastasis and progression (5–8). While many commonly used chemotherapeutic agents can eliminate tumor-associated myeloid cells, the killing effect of chemotherapy on myeloid cells is often transient and these cells eventually rebound after chemotherapy. Mounting evidence indicates that myeloid cells reemerged after chemotherapy contribute to tumor chemoresistance (9, 10).

The paradoxical effects of chemotherapy are exemplified by the alkylating agent cyclophosphamide (CTX). CTX is used, along with other chemotherapeutic agents, in the clinic to prepare patients with certain types of blood cancer for hematopoietic stem cell transplantation (HSCT) (11). CTX-based nonmyeloabaltive chemotherapy has also been widely used in adoptive T cell therapy (ACT) to precondition cancer patients prior to T cell infusion (12–14). These chemo-immunotherapies benefit from CTX’s debulking effect due to its direct cytotoxicity on fast-growing transformed cells, and the ability of CTX to incite antitumor immune responses by inducing immunogenic cell death, reducing Tregs and MDSCs, removing “cytokine sinks”, and creating space to facilitate T cell expansion (13, 14). On the other hand, it has been shown that CTX can condition a microenvironment receptive to tumor metastasis (14–16). We and others reported that CTX can induce immunosuppressive myeloid cells (17–21), which act to attenuate antitumor immune responses (22). Induction of tumor-promoting myeloid cells is not unique to CTX, a number of widely used anticancer drugs, including melphalan, doxorubicin and paclitaxel, are found to have similar effects (22–26). So far the ontogeny and molecular feature of these therapy-induced myeloid cells are poorly defined.

Cytotoxic chemotherapy causes cell death and bone marrow (BM) injury (27, 28). As a self-repairing mechanism in response to stress, dormant hematopoietic stem cells (HSCs) are rapidly recruited into the cell cycle to initiate hematopoietic recovery, including myelopoiesis, which is a regulated multistep process that leads to restoration of depleted cells of the myeloid lineage (29–31). During this process, HSCs give rise to common myeloid progenitors (CMPs). Granulocyte-macrophage progenitors (GMPs) derived from CMPs give rise to committed granulocytic precursors (GPs) and macrophage-dendritic cell progenitors (MDPs) (32, 33). A recent study demonstrated that in mice monocytes can derive independently from both GMPs and MDPs, and that GMP-derived monocytes acquire some characteristics of neutrophils (34). In a different study these neutrophil-like monocytes are found to exert immunoregulatory and tissue-reparative activities during systemic inflammation or tissue injury (35).

In this study, we conducted phenotypic and functional analyses and gene expression profiling to examine the impact of CTX on the myeloid cell compartment. Our work provides evidence that CTX-induced myeloid cell expansion is a default process intrinsic to hematopoietic recovery after nonmyeloablative chemotherapy, and that this process is subjected to regulation by DNA methyltransferase 1 (Dnmt1) and associated with chemotherapy-induced microbial translocation. During this process, the monocytes develop immunosuppressive capability and acquire a gene signature characteristic of neutrophil precursors. These findings may have implications for a broad range of chemo-immunotherapies involving the use of CTX.

## Materials and Methods

### Mice

Balb/c mice (CD45.2^+/+^) of 4 to 6 weeks of age were purchased from Charles River. The CD45.1^+/+^ mice on a Balb/c background and immunocompromised NOD-Scid IL2Rγ-null mice (NSG) mice were purchased from Jackson Laboratory. All mice were housed under specific pathogen-free (SPF) conditions by Laboratory Animal Services of the Augusta University. All animal experiments were approved by the Institutional Animal Care and Use Committee (IACUC) of Augusta University.

### Antibodies and Reagents

The following anti-mouse antibodies, including Lineage cocktail (17A2; RB6-8C5, RA3-6B2; Ter-119; M1/70); CD117 (cKit, 2B8), Ly-6A/E (Sca-1, D7), CD3 (145-2C11), CD19 (6D5), B220 (RA3-6B2), Ly-6C (HK1.4), Ly-6G (1A8), CD45.1 (A20), CD24 (M1/69), and CX3CR1 (SA011F11) were purchased from Biolegend. Ki-67-FITC staining set was purchased from BD Biosciences. Mouse CCR2 APC-conjugated antibody was purchased from R&D Systems. Anti-myeloperoxidase (MPO) antibody (2D4) FITC was purchased from Abcam. Intracellular fixation and permeabilization solution kit was purchased from BD. Violet cell proliferation kit were purchased from Thermo Fisher Scientific. Cyclophosphamide monohydrate (CTX) was purchased from Tokyo Chemical Industry. 5-azacytidine was purchased from Selleckchem. Anti-mouse TNFa (XT3.11) mAb was purchased from Bio X Cell. Mouse IL6 neutralization mAb was purchased from Biolegend.

### *In Vivo* Animal Treatments

Cyclophosphamide (CTX) was dissolved in PBS and was intraperitoneally injected to mice at 150 mg/kg. 5-azacytidine was injected to mice at 5 mg/kg following the specified schedule. All chemotherapy solutions were filtered through a 0.22 uM filter before injection. αTNFα or αIL6 mAb was given to mice by daily intraperitoneally injection for 5 days, starting from 1 day after CTX treatment. A cocktail of antibiotics (0.2 µg/ml of gentamicin, 0.15 µg/ml of ciprofloxacin, 2 mg/ml of streptomycin, and 1 mg/ml of bacitracin) was given to mice orally in drinking water 7 days prior to CTX treatment and maintained for the duration of the experiment as previously described (36).

### Cell Preparation and Flow Cytometry

Single-cell suspensions were prepared from spleen or bone marrow samples for flow cytometry analysis. Red blood cells were lysed by ACK lysing buffer. For surface molecule detection, cells were stained with fluorochrome-conjugated antibodies for 10 min at room temperature in the dark. To detect intracellular molecules, intracellular fixation and permeabilization kit (BD Biosciences) or transcription factor staining buffer set (eBioscience) was used following manufacturer’s instruction. All FACS data were acquired on a LSRII instrument (BD Biosciences) and analyzed using Flowjo software (Tree Star).

To isolate different subsets of myeloid cells, cells were stained with CD11b-FITC and Ly6C-PE/Cy7 mAbs and subjected to cell sorting on a FACSAria (BD Biosciences). The purity of sorted cells was usually greater than 95%.

### RNA Extraction and Quantitative Real-Time PCR (qRT-PCR)

Total RNAs from bone marrow aspirates or sorted monocytes were extracted using TRIzol Reagent (Thermo Fisher Scientific). 1 μg of RNA was used to generate cDNA using SuperScript III First-Strand Synthesis System (Thermo Fisher). qRT-PCR was performed using SYBR Green Mix (Bio-Rad), and cDNA amplification was performed on a BioRad iCycler equipped with an iCycler iQ Detection System. To verify that a single product was amplified, a melting curve was generated at the end of each run. The sequences of the primers used in this report can be found in published studies (37, 38). All primers were purchased from Integrated DNA Technologies Inc. β-actin was used to normalize target gene RNA expression levels. The comparative threshold cycle (*C*_t_) method was used to calculate amplification levels as specified by the manufacturer.

### *In Vitro* Suppression Assay

Spleen cells from normal Balb/c mice were labeled with 0.1 uM violet dye and seeded into a round-bottom 96-well plate (1 × 10^5^ cells/well in 200-µl medium), with or without the addition of 1 µg/ml of anti-CD3 (145-2C11) and 5 µg/ml of anti-CD28 (37.51). Varied numbers of sorted monocytes or neutrophils were added to culture. Cells were harvested at indicated time points for flow cytometry analysis.

### Cell Preparations for Microarray Analysis

7 days after CTX treatment, monocytes (CD11b^+^Ly6C^hi^) and neutrophils (CD11b^+^Ly6C^lo^) were flow-sorted from splenocytes. Myeloid cells from naïve Balb/c splenocytes were used as control. Total RNA was isolated using Invitrogen PureLink RNA mini kit according to manufacturer’s protocol. The concentration of isolated RNA was determined using a Nanodrop spectrophotometer (Thermo Scientific) and the quality of RNA was analyzed on a 2100 Bioanalyzer (Agilent Technologies, Santa Clara, CA). RNAs with an RNA integrity number (RIN) greater than 7.5 were used to generate sense strand cDNAs from polyadenylated mRNAs in 1 µg of the total RNA samples using GeneChip WT Expression kit (Affymetrix, Santa Clara, CA). The cDNAs were then fragmented and biotin-labeled using GeneChip WT Terminal Labeling kit (Affymetrix). The biotin-labeled cDNAs were hybridized onto a Mouse Gene 2.0ST array according to the manufacturer’s protocol. 16 h after hybridization, the arrays were washed and stained using Affymetrix GeneChip Fluidics Station 450 system. The stained arrays were scanned on an Affymetrix GeneChip Scanner 3000 at the Integrated Genomic Core, Augusta University, GA. The expression data were obtained in the form of CEL files.

### Bioinformatics Analysis

The CEL files were analyzed in R version 3.6.3 using a workflow developed by Bernd Klaus and Stefanie Reisenauer based on Bioconductor Packages. The array was normalized using Robust Multi-array Average (RMA) method (Oligo v 1.48.0). The differentially expressed genes were identified using limma package (v3.40.6). For multiple testing correction, the FDR procedure developed by Benjamini et al. was used (39). To derive the neutrophil-like monocytes gene signature, the scRNAseq data set (GSE104478) was downloaded from Gene Expression Omnibus (GEO) and analyzed using Seurat v3.1.5. The single cell cluster consisting neutrophil-like monocytes was determined by multiple genes typically expressed in neutrophil precursors, such as Elane, Mpo, Serpinb1a, Ctsg and Prtn3. A gene signature representing the neutrophil-like monocyte cluster was identified by FindMarkers function in Seurat. A subset of 650 genes with absolute log2FC > 0.5849 (1.5 fold change) were selected as a custom gene set for comparison with the Affymetrix array data using GSEA method in clusterProfiler v3.12.0 (40). Clustering and heatmaps were generated using ComplexHeatmap v2.0.0.

### Statistical Analysis

Data were analyzed using Prism 7.0 (GraphPad Software, Inc.). For comparison between two groups, the statistical significance of the results was determined using unpaired two-tailed Student’s *t* test. One-way ANOVA was used to determine statistical differences between three of more groups. *P* values less than 0.05 were considered statistically significant.

## Results

### Myeloid Cell Expansion After CTX Is Inherent to the Hematopoietic Recovery Process in Host Response to Non-Myeloablative Chemotherapy

**Figure 1:**
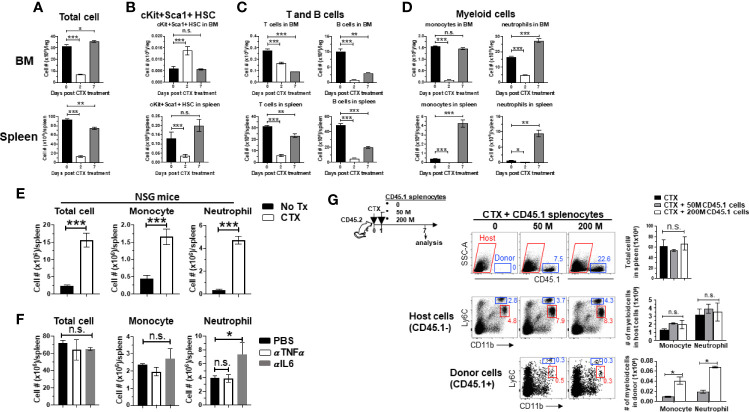
Myeloid cell expansion after CTX is inherent to the hematopoietic recovery process in host response to non-myeloablative chemotherapy. (**A** to **D**) The kinetics of immune cell recovery in mice after CTX treatment. Naïve Balb/c mice were untreated or treated with 150 mg/kg CTX. At the indicated time points, cells from spleens and BMs were enumerated and analyzed for the frequencies of HSCs (Lin^-^cKit^+^Sca1^+^), B cells (CD19^+^B220^+^), T cells (CD3^+^), monocytes (CD11b^+^Ly6C^hi^), and neutrophils (CD11b^+^Ly6C^lo^) by flow cytometry. The numbers of total cells **(A)**, HSCs **(B)**, T and B cells **(C)**, monocytes and neutrophils **(D)** in spleens and BMs are summarized in the bar graphs. The formula for calculating the cell number of each specified cell population is: total cell number x percent of specific cell population. Results are shown as mean ± SD of at least three mice per group at each time point. **(E)** CTX-driven myeloid cell expansion in mice is independent of the adaptive immune system. NSG mice were not treated or treated with CTX. 7 days later, spleen cells were enumerated and stained for CD11b and Ly6C to determine the absolute number of monocytes and neutrophils. Results are summarized in bar graphs and shown as mean ± SD of three mice each group. **(F)** Neutralization of TNFα or IL6 does not affect CTX-induced myeloid cell expansion. Naïve Balb/c mice were treated with CTX. A cohort of mice were further treated with TNFα or IL6 neutralizing monoclonal antibody (100 µg/injection in 200 ul PBS) by daily intraperitoneally injection for 5 days, starting 1 day after CTX treatment. On day 7 after CTX treatment, spleens were collected for flow cytometry analysis. The absolute number of each myeloid cell subset in the spleens from the mice with different treatments are summarized in bar graphs. Data are shown as mean ± SD with at least three mice per group. **(G)** Filling the “space” created by CTX-induced lymphodepletion with bystander cells does not affect the expansion of the endogenous myeloid cells. Following the timeline depicted in the schema, naïve Balb/c (CD45.2^+/+^) mice were treated with CTX followed by adoptive transfer of increasing numbers of splenocytes derived from CD45.1^+/+^ mice the next day. 7 days after CTX treatment, spleens were collected and processed for flow cytometry analysis. Representative dot plots shown in the top panel indicate the presence of the CD45.1+ bystander donor cells. Numbers in dot plots represent the frequencies of donor cells in total splenocytes. The absolute numbers of total spleen cells shown as mean ± SD are summarized in the bar graph on the right. The frequencies of monocytes and neutrophils in the host (CD45.1^-^, middle panel) and donor (CD45.1^+^, bottom panel) splenocytes are shown in representative dot plots. The absolute numbers of monocytes and neutrophils in the host or donor splenocytes are summarized in bar graphs shown as mean ± SD of four samples per group. n.s., not significant, *p < 0.05, **p < 0.01, ***p < 0.001.

We previously showed that myeloid cell expansion is observed in mice treated with CTX in the dose rang of 100–300 mg/kg (22), which is lymphodepleting but nonmyeloablative. Others have reported that CTX given in this dose range can induce hematopoietic stem cells (HSC) proliferation and mobilization in mice (30, 41). Based on these data, we posited that the observed myeloid cell expansion after CTX is the result of hematopoietic recovery in response to bone marrow stress inflicted by nonmyeloablative chemotherapy. To test this, we examined the changes in the number of HSCs and the major immune cell populations, including T cells, B cells and myeloid cells, in the BM and spleen after CTX treatment. There was a drastic reduction in cellularity in both BM and spleen 2 days after CTX (**Figure 1A**). Notably, at this time point the number of lineage^-^Sca1^+^cKit^+^ (LSK) HSCs in the BM increased significantly (**Figure 1B**), while the numbers of T, B and myeloid cells experienced sharp reduction in the BM and spleen (**Figures 1C, D**). Moreover, the surge of LSK HSCs was restricted to the BM as LSK HSCs in the spleen declined on day 2 (**Figure 1B** bottom). By day 7, the total cell numbers in the BM and spleen have returned to the baseline levels (**Figure 1A**). The numbers of LSK HSCs contracted in the BM but increased in the spleen after day 2 and have returned to their respective baselines by day 7 (**Figure 1B**), suggesting the migration of HSCs from the BM to the periphery. To address whether local proliferation of splenic LSKs contributed to the LSK rebound in the spleen on day 7, splenocytes from CD45.1+ mice were transferred to PBS or CTX-treated mice (**Supplementary Figure 1** schema). Donor-derived LSKs were predominantly found in the spleen, and their numbers were comparable in PBS versus CTX-treated mice (**Supplementary Figure 1**), arguing against the likelihood of local expansion of spleen-resident LSKs. By day 7, the numbers of T and B cells in the BM and spleen have rebounded but have not reached their baseline levels (**Figure 1C**). In contrast, the numbers of myeloid cells, including monocytes and neutrophils, have either reached (in the BM) or significantly surpassed (in the spleen) the pre-treatment levels by day 7 (**Figure 1D** last column). The results indicate that HSCs are rapidly mobilized to the BM to start myelopoiesis in response to myelodepletion caused by chemotherapy, and this hematopoietic recovery process leads to a net increase in the number of myeloid cells in the periphery prior to the restoration of lymphocytes.

Some T cell-derived cytokines, including GM-CSF and TNFα, can promote myeloid cell expansion (42, 43). To examine whether the host T cells play a role in modulating myeloid cell expansion, immunodeficient NOD-Scid IL2Rγ-null (NSG) mice, which lack T, B and NK cells, were treated with CTX and enumerated for myeloid cells (monocytes and neutrophils) in the spleens 7 days later. **Figure 1E** shows that myeloid cell expansion was also observed in CTX-treated NSG mice, suggesting that myeloid cell expansion after chemotherapy is not influenced by the adaptive immune system. We also found that administration of neutralizing antibodies against IL-6 or TNFα, two cytokines known to regulate myeloid cell development and maturation (44, 45), did not reduce CTX-driven myeloid cell expansion in wild-type mice (**Figure 1F**), although the possible role of other inflammatory cytokines in myeloid cell expansion in this setting remains to be determined. It is known that CTX-induced lymphodepletion can create “space” and increased availability of growth factors, which are known to promote the expansion of adoptively transferred T cells (12–14). This prompted us to examine whether these features associated with lymphodepleting CTX contributed to the expansion of the myeloid cells. To address this, naïve mice (CD45.2^+^) were treated by CTX, followed by adoptive transfer of increasing doses of splenocytes from CD45.1^+^ mice to fill the “space” induced by CTX (**Figure 1G** schema). We found that the total cell number in the spleen of a mouse remained steady regardless of whether or not the mouse had received exogenous bystander splenocytes (**Figure 1G** top panel), likely due to homeostasis of cell number. Moreover, CTX-induced myeloid cell expansion was not attenuated in the presence of bystander cells (**Figure 1G** middle panel). Although infusion of more bystander cells resulted in more donor-derived myeloid cells, the numbers of donor-derived monocytes and neutrophils were negligible (50-fold less) compared to host-derived monocytes and neutrophils (**Figure 1G** bottom panel). Altogether, these results suggest that myeloid cell expansion after CTX is intrinsic to the hematopoietic recovery process after non-myeloblative chemotherapy, and this hardwired feature is independent of the influence of the adaptive immune system, as well as the availability of space and growth factors in the post-chemotherapy environment.

### Monocytes in the Bone Marrow and Spleen Acquire Immunosuppressive Activity After CTX Treatment

**Figure 2:**
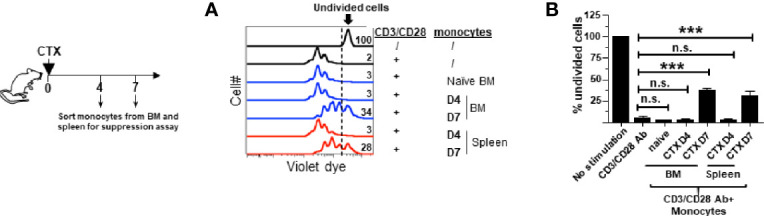
Monocytes in the spleens and bone marrows acquire suppressive activity after CTX treatment. Following the timeline depicted in the schema, naïve Balb/c mice were treated with CTX. At the indicated time points (day 4 and 7 after CTX treatment), spleens and bone marrows were collected and stained for CD11b and Ly6C. Monocytes (CD11b^+^Ly6C^hi^) and neutrophils (CD11b^+^Ly6C^lo^) were flow-sorted for *in vitro* T-cell suppression assay. Monocytes from the BM of untreated naïve mice were included as a control. Spleen cells from naïve Balb/c were labeled with violet dye, and the T cells within the splenocytes served as responder cells. Responder cells were mixed with equal numbers of sorted myeloid cells and stimulated with aCD3/aCD28 mAbs. After 3 days in culture, cells were analyzed by flow cytometry. **(A)** The proliferation of responder T cells was evaluated by violet dye dilution. The numbers in the histogram overlay indicate the percentage of undivided cells under each condition. **(B)** The frequencies of undivided responder T cells under each culture condition are summarized in bar graph and shown as mean ± SD of triplicate cultures. Data shown are representative of two independent experiments. n.s. not significant, ***p < 0.001.

We previously reported that the monocytes recovered from the spleens of CTX-treated mice exhibited immunosuppressive activity while their counterparts from naïve mice were not immunosuppressive (22), suggesting that immunosuppression is an acquired property for monocytes expanded after CTX treatment. We asked when after CTX treatment monocytes acquire suppressive activity, and whether monocytes in the bone marrow also become immunosuppressive. To address these questions, spleen cells and bone marrow aspirates from mice were harvested at different time points after CTX treatment. Monocytes (CD11b^+^Ly6C^hi^) were flow-sorted and used for *in vitro* T-cell suppression assay. **Figure 2** shows that monocytes from the BM of untreated naive mice (D0) or mice treated with CTX 4 days ago (D4) did not suppress T cell proliferation, whereas BM monocytes isolated from mice 7 days (D7) after CTX treatment exhibited immunosuppressive activity. Similar results were observed for monocytes isolated from the spleens of CTX-treated mice. The results suggest that acquisition of immunosuppressive capacity by monocytes after CTX is a process that occurs systemically and takes approximately 7 days.

### Monocytes in CTX-Treated Mice Retain Suppressive Activity Even After Myeloid Cell Homeostasis Is Restored

**Figure 3:**
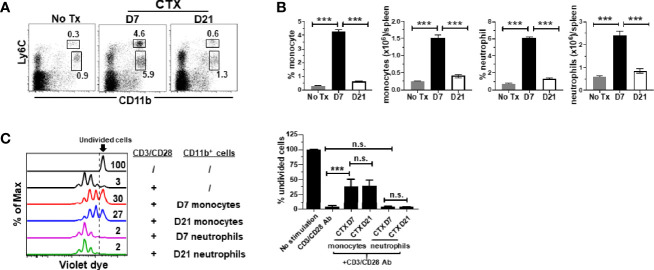
Monocytes from CTX-treated mice retain suppressive activity even after myeloid cell homeostasis is restored. CTX was given to naïve Balb/c mice either 7 or 21 days prior to cell harvest. Spleen cells were collected and stained for CD11b and Ly6C. **(A)** Representative dot plots are shown, and the numbers indicate the frequencies of gated populations. The results are summarized in bar graphs with at least three mice each group **(B)**. The formula for cell number calculation is: total cell number x percent of specific myeloid cell subset. **(C)** In vitro suppression assay. In vitro suppression culture was setup as described in **Figure 2**. Equal number of responder cells and sorted myeloid cells were used. Proliferation of responder T cells was evaluated by violet dilution. Numbers in histogram indicate the percent of undivided responder T cells. The frequencies of undivided responder T cells under each culture condition shown as mean ± SD of triplicate cultures are summarized in the bar graph on the right. Data shown are representative of two independent experiments. n.s., not significant, ***p < 0.001.

It is unknown whether the acquired suppressive activity of monocytes after CTX is a transient or persistent feature. Our previous studies showed that myeloid cells underwent expansion after CTX treatment, reaching peak on day 7 followed by contraction, and the level of myeloid cells returned to the baseline after 14 days (22). We chose to examine the suppressive activity of monocytes isolated 21 days after CTX, when myeloid cell homeostasis has been restored. We confirmed that 21 days after CTX, the frequencies and absolute cell numbers of myeloid cells, including both monocytes and neutrophils, were undisguisable from those in naïve mice (**Figures 3A, B**). Monocytes and neutrophils were flow-sorted and subjected to *in vitro* suppression assay. Notably, monocytes from mice 21 days after CTX treatment were equally suppressive as those from mice 7 days after CTX treatment (**Figure 3C**). As controls, neutrophils recovered at both time points were non-suppressive. The data indicate that monocytes repopulated after CTX retain immunosuppressive capacity for an extended period of time.

### Repeated Administration of CTX Leads to Accumulation of Myeloid Cells That Contain Immunosuppressive Monocytes

**Figure 4:**
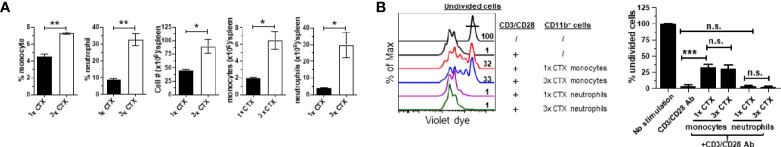
Repeated CTX administration results in enhanced expansion of myeloid cells. Mice were injected with CTX weekly for 3 weeks (3x-CTX). 7 days after the last CTX injection, spleen cells were harvested for analysis. Mice receiving one dose CTX (1x-CTX) were used for comparison. **(A)** Frequencies and numbers of monocytes and neutrophils in spleens are summarized in bar graphs with three mice each group. **(B)** *In vitro* suppression assay. Violet dye-labeled responder T cells were mixed with equal numbers of sorted myeloid cells derived from mice receiving either one or three doses of CT and stimulated with aCD3/aCD28 mAbs. Proliferation of T cells was measured by violet dye dilution. The rightmost peak represents undivided T cells. Numbers in histogram indicate the percent of undivided responder T cells. The frequencies of undivided responder T cells under each culture condition are summarized in bar graph and shown as mean ± SD of triplicate cultures. Data shown are representative of two independent experiments. n.s., not significant, *p < 0.05, **p < 0.01, ***p < 0.001.

In the clinic chemotherapeutic agents are often given to patients in repeated cycles. This prompted us to examine the impact of repeated CTX administration on the expansion of myeloid cells and the suppressive activity of monocytes. To this end, mice were either injected with a single dose of CTX (1x CTX), or three doses of CTX given in 3 weeks (3x CTX). 7 days after the last dose of CTX, mice were sacrificed to analyze the frequencies and absolute numbers of monocytes and neutrophils in the spleens. **Figure 4A** shows that three doses of CTX led to further accumulation of myeloid cells including both monocytes and neutrophils, compared to single dose of CTX. Furthermore, the monocytes from 3x CTX-treated mice exhibited comparable suppressive capability as those from 1x CTX-treated mice, whereas neutrophils remained to be non-suppressive under either condition (**Figure 4B**).

### Microarray Analysis Reveals the Emergence of Neutrophil-Like Monocytes in CTX-Treated Mice

**Figure 5:**
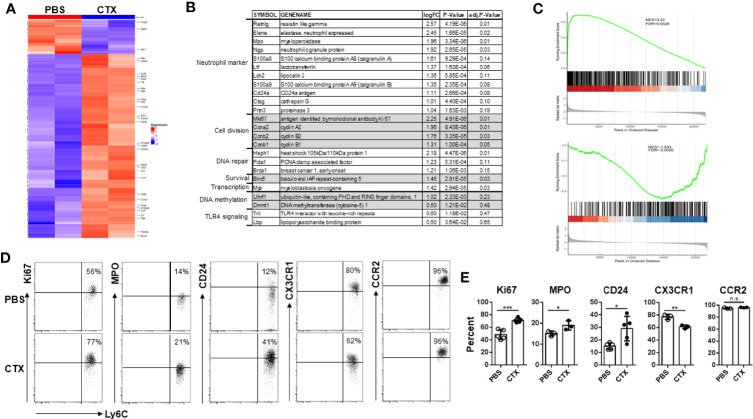
Microarray analysis reveals the emergence of neutrophil-like monocytes in CTX-treated mice. **(A)** Heatmap showing the differential gene expression profiles of monocytes from CTX-treated vs. control mice. 7 days after CTX treatment, CD11b^+^Ly6C^hi^ monocytes were flow-sorted from splenocytes. Monocytes from untreated naïve Balb/c splenocytes were used as control. Total RNA was isolated and subjected to microarray analysis using Affymetrix GeneChip. 96 differentially expressed genes (absolute logFC ≥ 1 and FDR <0.2) identified by limma package were used for Hierarchical clustering. **(B)** A list of candidate genes significantly upregulated in monocytes from CTX-treated mice compared to monocytes from untreated mice. Genes with similar functions are grouped together. **(C)** GSEA results demonstrating enrichment of gene signature characteristic of neutrophil-like monocytes in differentially expressed genes induced by CTX-treatment. Genes up- or down-regulated in neutrophil-like monocytes were derived from a publicly available scRNAseq dataset (GSE104478) and used as separate gene sets for comparison with rank sorted gene expression (logFC) in monocytes between CTX-treated vs. control mice. **(D)** Naïve Balb/c mice were untreated or treated with 150 mg/kg CTX. 7 days after treatment, spleens were processed and the differential expressions of a panel of molecules in monocytes from untreated and CTX-treated mice were confirmed by flow cytometry. Numbers in dot plots indicate percentage of cells in the corresponding quadrant. The results of flow cytometry analysis are summarized in bar graph **(E)**. The results are shown as mean ± SD with at least three mice per group. n.s., not significant, *p < 0.05, **p < 0.01, ***p < 0.001.

Acquisition of immunosuppressive capacity by monocytes after CTX implied gain of function in these cells, which was likely associated with changes in gene expression. Thus we performed microarray analysis to examine the impact of CTX on global gene expression in monocytes. Using absolute log2 fold change (logFC) ≥ 1 and false discovery rate (FDR) < 0.2 as cutoff criteria, we identified 96 up-regulated and 10 down-regulated genes in CTX-monocytes as compared to naïve monocytes (**Figure 5A**). **Figure 5B** lists a selected panel of genes which showed significantly elevated expressions in monocytes from CTX-treated mice compared to monocytes from naive mice. Notably, multiple granules typically expressed in neutrophil precursors, including Elane, Mpo, Ngp, Ltf, Lcn2, Ctsg and Prtn3, were upregulated in monocytes from CTX-treated mice. These molecules are mainly primary and secondary granules expressed in early neutrophil precursors, such as premyelocytes and myelocytes (46, 47). In addition, CD24, a typical neutrophil marker, was also upregulated in monocytes from CTX-treated mice. It is worth noting that a number of cell-cycle genes, including Ccna2, Ccnb1, and Ccnb2, were also significantly upregulated in monocytes from CTX-treated mice, suggesting that these monocytes were active in cell division. Consistent with an active cell cycle status, the cell proliferation marker Mki67 was elevated in monocytes from CTX-treated mice. The transcripts of several genes involved in DNA repair, including Hsph1, Pclaf and Brca1, were upregulated along with the pro-survival gene Birc5, in line with the occurrence of myelopoiesis in response to chemotherapy. Yanez et al. recently reported the identification of GMP-derived neutrophil-like monocytes in mice *via* single-cell RNA sequencing analysis (scRNAseq) (34). We re-analyzed their publicly available scRNAseq dataset (GSE104478) and found that the transcriptomic signature of those neutrophil-like monocytes consists 650 genes with absolute logFC > 0.5849 (1.5 fold change) (**Supplementary Table I**). We performed gene set enrichment analysis (GSEA) to examine how the differentially expressed genes in our microarray dataset correlate with the gene signature of neutrophil-like monocytes. We found that genes up-regulated in neutrophil-like monocytes were up-regulated in monocytes derived from CTX-treated mice (**Figure 5C** top plot), and genes down-regulated in neutrophil-like monocytes were also down-regulated in monocytes derived from CTX-treated mice (**Figure 5C** bottom plot). The results support the notion that neutrophil-like monocytes arise among the myeloid cells repopulated in CTX-treated mice. We further confirmed by flow cytometry the differential expression of some of the molecules, including Ki67, MPO, and CD24 (**Figures 5D, E**). On the opposite, the level of CX3CR1, a chemokine receptor expressed by monocytes, was reduced in monocytes from CTX-treated mice. Interestingly, the levels of CCR2, another chemokine receptor highly expressed by monocytes, remained the same in monocytes before and after CTX treatment, suggesting that alteration in gene expression in monocytes is selective rather than ubiquitous. Altogether, our data indicate that the monocytes emerging after CTX treatment acquire a gene signature characteristic of neutrophil precursors.

### CTX-Driven Myeloid Cell Expansion Is Diminished by DNMT1 Inhibitor 5-Azacitidine

**Figure 6:**
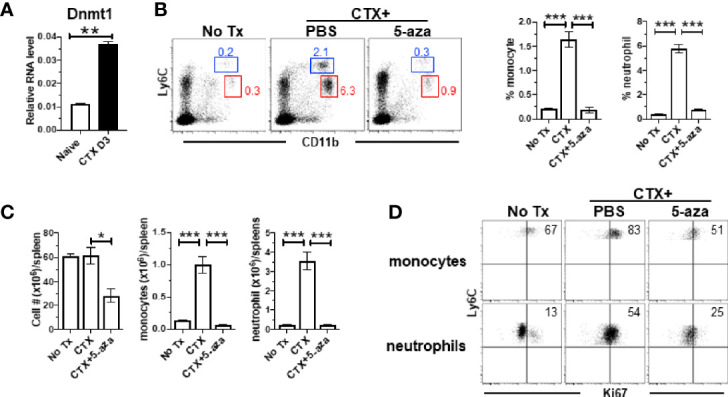
CTX induces DNMT1 in BM and DNMT1 inhibitor 5-azacytidine abrogates myeloid cell expansion. **(A)** CTX induces DNMT1 expression in BM. Naïve Balb/c mice were untreated or treated with CTX. 3 days later, BM cells were subjected to qRT-PCR for DNMT1. The DNMT1 expression is normalized to β-actin. **(B)** DNMT1 inhibition abrogates CTX-induced myeloid cell expansion. Naïve Balb/c mice were treated with CTX, followed by ip injection of 5-aza (100ug ip on d1, 3, 5). On day 7, spleen cells were harvested and stained for CD11b and Ly6C. Representative dot plots are shown, and the numbers indicate the frequencies of gated populations. The frequencies of each myeloid subset are summarized in bar graphs with at least three mice each group. **(C)** The bar graphs summarize the numbers of monocytes and neutrophils under the indicated condition with at least three mice per group. **(D)** Expression profiles of Ki67 in monocytes and neutrophils from the mice with the indicated treatments. Numbers in dot plots indicate percentage of cells in the corresponding quadrant. *p < 0.05, **p < 0.01, ***p < 0.001.

Our microarray data showed that Ubiquitin-like with PHD and ring finger domains 1 (Uhrf1) and DNA methyltransferase 1 (Dnmt1) were both upregulated in monocytes from CTX-treated mice, though their adjusted P values did not reach the cutoff criterion (**Figure 5B**). Uhrf1 is known to bind to hemimethylated DNA and recruit DNMT1 to epigenetically regulate gene expression (48–50). We confirmed by quantitative RT-PCR that Dnmt1 was significantly increased in the BM cells from CTX-treated mice (**Figure 6A**). We examined whether targeting DNMT1 with 5-azacitidine (5-aza), a potent DNMT1 inhibitor, would affect post-CTX myeloid cell expansion in mice. **Figure 6B** shows that 5-aza markedly reduced the frequencies of both monocytes and neutrophils in the spleens of CTX-treated mice. 5-aza also led to reduced cellularity in the spleens of CTX-treated mice, resulting in significant reductions in the numbers of monocytes and neutrophils (**Figure 6C**). The reduction of myeloid cells in CTX-treated mice by 5-aza might be due to inhibition of myeloid cell proliferation. To test this, we examined the levels of Ki67 in monocytes and neutrophils in mice that were either untreated, or CTX-treated, or CTX-treated followed by 5-aza injection. In steady state, monocytes were more proliferative than neutrophils (**Figure 6D** left panel). Both myeloid subsets showed increased proliferative capacity after CTX treatment (**Figure 6D** middle panel), indicating ongoing myelopoiesis during this time period. 5-aza administration markedly diminished CTX-driven proliferation of monocytes and neutrophils (**Figure 6D** right panel). As a control, we treated mice with CTX followed by nanaomycin A, a selective DNMT3B inhibitor. Similar to 5-aza, addition of nanaomycin A after CTX reduced the cellularity in the spleen (**Supplementary Figure 2A**). However, unlike 5-aza, nanaomycin A did not change the frequencies of monocytes and neutrophils in CTX-treated mice (**Supplementary Figure 2B**), even though the absolute numbers of these myeloid cells decreased as the result of reduction in total cell number (**Supplementary Figure 2C**). The data suggest that DNMT3B inhibition impairs hematopoiesis but does not directly affect myelopoiesis, whereas Dnmt1 is uniquely involved in regulating myelopoiesis after CTX.

### Antibiotics Administration Reduces CTX-Driven Myeloid Cell Expansion and the Suppressive Activity of Monocytes

**Figure 7:**
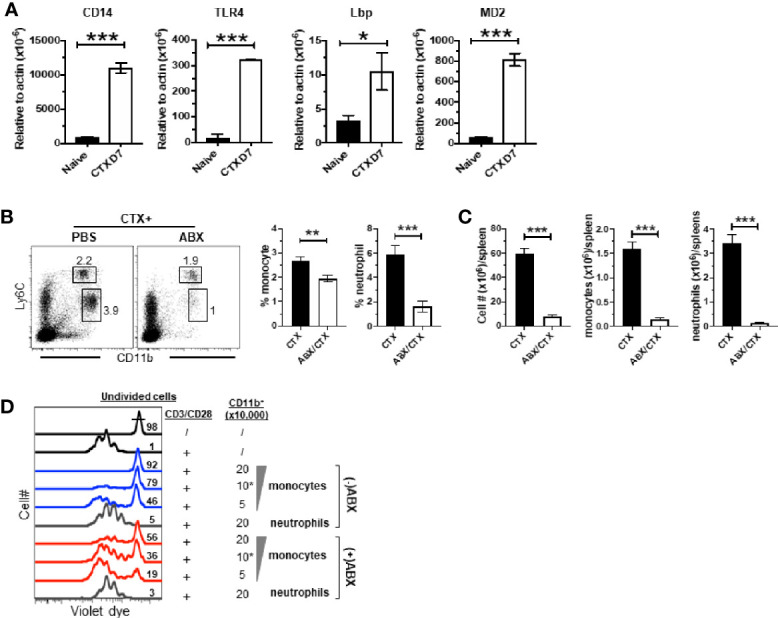
Antibiotics administration reduces CTX-driven myeloid cell expansion and the suppressive activity of monocytes. **(A)** CTX activates TLR4 signaling pathway in monocytes. Naïve BALB/c mice were treated with CTX. On day 7, CD11b^+^Ly6C^hi^ monocytes were flow-sorted from the splenocytes recovered from CTX-treated mice. Monocytes from the splenocytes from naïve BALB/c mice were used as control. Total RNA was extracted from sorted monocytes and subjected to qRT-PCR to evaluate the mRNA levels of the indicated genes. The target gene transcripts are normalized to β-actin. **(B)** Antibiotics administration inhibits CTX-induced myeloid cell expansion. Normal drinking water (H_2_O) or antibiotics-containing water (ABX) was provided to mice 7 days before CTX treatment, and maintained for the duration of the experiment. 7 days after CTX treatment, spleen cells were processed and stained for CD11b and Ly6C. Representative dot plots are shown, and the numbers indicate the frequencies of gated populations. The frequencies of each myeloid subset are summarized in bar graphs with at least three mice each group. **(C)** The bar graphs summarize the numbers of monocytes and neutrophils in mice receiving CTX or CTX+ABX treatment. **(D)** ABX administration reduces the suppressive activity of monocytes from CTX-treated mice. Violet dye-labeled responder T cells were mixed with indicated numbers of sorted myeloid cells derived from mice receiving either CTX only (-ABX) or CTX+ABX (+ABX), and stimulated with aCD3/aCD28 mAbs. Proliferation of T cells was measured by violet dye dilution. Numbers in histogram indicate the percent of undivided responder T cells. *p < 0.05, **p < 0.01, ***p < 0.001.

It is increasingly appreciated that the intestinal microbiota can influence the composition, expansion and function of myeloid cells in the host (44, 45). CTX is known to cause translocation of intestinal bacteria (36, 51, 52). We noticed that in our microarray data the transcripts of TLR4 interactor with leucine rich repeats (Tril), a component of the TLR4 complex, and Lipopolysaccharide (LPS) binding protein (Lbp) were both modestly upregulated (~1.5-fold) in monocytes from CTX-treated mice though their adjusted P values did not reach the cutoff criterion (**Figure 5B**), suggesting that the TLR4 signaling pathway may be activated in monocytes recovered from CTX-treated mice. Indeed, real-time quantitative RTPCR (qRTPCR) revealed that the monocytes from CTX-treated mice exhibited significantly higher levels of transcripts of CD14, TLR4, Lbp, and MD2, the major components of the LPS receptor complex (**Figure 7A**). This result raised the possibility that translocation of intestinal microbiota may contribute to the observed myeloid cell expansion after CTX treatment. To test this, mice were given either normal drinking water or water containing a broad-spectrum antibiotic cocktail (ABX) before and after CTX treatment. **Figure 7B** shows that antibiotics administration reduced the frequencies of monocytes and neutrophils in the spleens of CTX-treated mice. Antibiotics also reduced the spleen cellularity, resulting in severely reduced number of monocytes and neutrophils in CTX-treated mice (**Figure 7C**). We next conducted *in vitro* suppression assay to examine the impact of ABX on the suppression activity of monocytes in CTX-treated mice. To compare the suppression potency of monocytes isolated from ABX-treated versus ABX-naïve mice, escalating doses of monocytes were co-cultured with responder T cells in the presence of stimulation by plate-coated aCD3 and aCD28 antibodies. **Figure 7D** shows that monocytes from ABX-naïve mice inhibited T cell proliferation in a dose-dependent manner, whereas monocytes from ABX-treated mice exhibited compromised suppressive ability at each dose tested. For example, 50,000, 100,000, and 200,000 monocytes from ABX-naïve mice prevented 46, 79, and 92% of T cells from undergoing cell division, respectively. In comparison, the percent of undivided T cells dropped to 19, 36, and 56%, respectively, when the same numbers of monocytes from ABX-treated mice were used. In contrast, neutrophils remained to be non-suppressive regardless of ABX usage. Our data suggest that microbial translation regulates CTX-driven myeloid cell expansion and acquisition of immunosuppressive activity by monocytes.

## Discussion

CTX-induced myeloid cell expansion has been well-documented (17–22). The current study further reveals the heterogeneity of the myeloid cells, especially the monocytes, in the post-CTX window. We showed that after nonmyeloablative chemotherapy with CTX, a subset of the newly expanded monocytes acquired a gene signature characteristic of neutrophil precursors. In line with the changes in transcription profile, some monocytes gained expression of CD24, which a marker for neutrophil precursors at the myelocyte stage (53). Chemokine receptors CCR2 and CX3CR1 are highly expressed by monocytes and known to regulate the trafficking of monocytes, with CCR2 promoting the release of monocytes from BM to peripheral tissues while CX3CR1 facilitating monocyte BM retention (54). We showed that the monocytes repopulating after CTX downregulated CX3CR1 while maintaining high level of CCR2, consistent with the notion that there is increased need of myelopoiesis after chemotherapy to replenish the depleted myeloid cells.

During myelopoiesis, HSCs give rise to CMPs which subsequently generate GMPs. GMPs give rise to committed granulocytic precursors (GPs), which differentiate into neutrophils, and macrophage-dendritic cell progenitors (MDPs), which differentiate into monocytes, DCs and macrophages (32, 33). A recent study demonstrated that mouse MDPs arose from CMPs independently of GMPs, and that GMPs and MDPs produce monocytes with distinct features (34). GMPs produce neutrophils along with a subset of neutrophil-like monocytes, whereas MDPs give rise to monocytes that can further develop into dendritic cells. The neutrophil-like monocytes express higher levels of primary granules Elane, Prtn3 and Ctsg compared to MDP-derived monocytes. Ikeda et al. reported that a subset of monocytes expanded during the recovery phase of systemic inflammation or tissue injury exhibit some neutrophil characteristics and exert immunoregulatory and tissue-reparative activities (35). We showed here that a subset of the monocytes repopulated in mice after CTX shared similar transcriptomic profiles as those reported neutrophil-like monocytes, suggesting that these cells develop through the CMP-GMP-monocyte lineage. In the published studies, the emergence of neutrophil-like monocytes is driven by microbial invasion such as LPS administration (34, 35). Our results that antibiotics administration diminished the number and immunosuppressive activity of monocytes after CTX also suggest a role of chemotherapy-induced microbial translocation in the rise of neutrophil-like monocytes. It is worth noting that a subset of monocytes capable of giving rise to polymorphonuclear MDSCs (PMN-MDSCs), termed monocyte-like precursors of granulocytes (MLPGs), has been identified in tumor-bearing mice and patients with cancer (55). Although both neutrophil-like monocytes and MLPGs are descendants of GMPs, the etiologies of these two populations seem to differ. The rise of MLGPs is driven by abnormal myelopoiesis in the face of tumor progression, whereas the emergence of neutrophil-like monocytes appears to be the default host response to microbial invasion and tissue injury. Given the frequent use of chemotherapy in cancer treatment, there is possibility that chemotherapy-induced neutrophil-like monocytes and tumor-induced MLPGs may co-exist in patients. Whether and how these cells interact with each other and impact host response to chemotherapy await further investigation.

It is interesting that only CTX-induced monocytes are immunosuppressive while neutrophils are not. It remains to be determined whether the immunosuppressive effect of monocytes observed in our study can be solely attributed to the neutrophil-like subset. Neutrophils are the most abundant leukocytes in the circulation and the first line of defense against pathogens. Activated neutrophils act to eliminate invading microorganisms through phagocytosis and intracellular degradation, release of granules and reactive oxygen species (ROS), and formation of neutrophil extracellular traps (NETs) (56). The number and function of neutrophils need to be controlled to avoid causing collateral damages to self-tissues after clearance of pathogens. Grainger et al. reported that during acute mucosal infection of mice with Toxoplasma gondii, inflammatory monocytes acquire a regulatory phenotype and effectively suppress neutrophil-mediated pathology (57). Similarly, immunosuppressive monocytes may act as rheostats modulating the activity of neutrophils during myelopoiesis after nonmyeloablative chemotherapy.

Previous studies have implicated DNMT1 as an important mediator essential for HSC self-renewal, trafficking and myeloid lineage differentiation (58, 59). Mice with DNMT1-deficiency in HSCs exhibit defects in myeloid lineage differentiation (58). Several studies reported that the DNA methylatransferase inhibitor 5-aza can reduce the presence of tumor-induced and CTX-induced MDSCs in mice (60, 61). In the current study, we showed that epigenetic modifiers Dnmt1 and Uhrf1 were both upregulated in the monocytes recovering after CTX, suggesting that DNA methylation is involved in chemotherapy-induced myelopoiesis. In addition, we showed that 5-aza treatment primarily reduced the highly proliferative (Ki67+) monocytes and neutrophils in the post chemotherapy window (**Figure 6D**). Our results suggest that the substantial reduction of myeloid cells by 5-aza may occur at two levels: by disrupting the epigenetic regulation in HSCs during myelopoiesis and by direct killing of proliferative myeloid cells.

In summary, our study sheds new light on the differentiation, heterogeneity, and function of myeloid cells repopulating after chemotherapy. Our findings may guide the design of novel strategies targeting myeloid cells to improve the effectiveness while reducing the toxicity of the conditioning regimens, thereby enhancing the efficacy of subsequent cancer therapies such as HSCT or ACT.

## Data Availability Statement

The raw data supporting the conclusions of this article will be made available by the authors, without undue reservation.

## Ethics Statement

The animal study was reviewed and approved by Augusta University Institutional Animal Care and Use Committee (IACUC).

## Author Contributions

Z-CD performed research, analyzed data and wrote the paper. NA assisted experiments. LB advised study design and edited the paper. HS performed bioinformatics analyses and wrote the paper. GZ conceived the study, provided funding, designed research, analyzed data and wrote the paper. All authors contributed to the article and approved the submitted version.

## Funding

This work was supported by National Institutes of Health grants 1R01CA215523 and 1R01CA238514 to GZ. and 1R03CA212829 to HS.

## Conflict of Interest

The authors declare that the research was conducted in the absence of any commercial or financial relationships that could be construed as a potential conflict of interest.

## Supplementary Material

Supplementary Figure 1 Splenic HSCs do not undergo local expansion in the spleens of CTX-treated mice. Following the timeline depicted in the schema, naïve BALB/c (CD45.2^+/+^) mice were treated with PBS or CTX (150 mg/kg). The next day, a total of 50 million splenocytes derived from CD45.1^+/+^ mice were intravenously injected into each recipient. 7 days after PBS or CTX treatment, spleens and BMs were collected and processed for flow cytometry analysis. The numbers of Lin^-^cKit^+^Sca-1^+^ HSCs in donor cells (CD45.1^+^) shown as mean ± SD were summarized in bar graphs with three mice in each group. n.s. not significant.

Supplementary Figure 2 DNMT3b inhibition does not impair CTX-driven myelopoiesis. Naïve BALB/c mice were treated with CTX. At indicated time points (day 1, 3, 5 after CTX), a cohort of mice were ip injected with nanaomycin A (200 µg/injection). 7 days after CTX treatment, spleen cells were harvested for analysis. (A) The total spleen cell numbers under each condition are summarized in bar graph with at least three mice per group. (B) Representative dot plots shown indicate the presence of myeloid cell subsets in spleen, and the numbers indicate the frequencies of gated populations. The frequencies of each myeloid subset are summarized in bar graphs with at least three mice each group. (C) The bar graphs summarize the numbers of monocytes and neutrophils under the indicated condition with at least three mice per group. n.s. not significant, * *p* < 0.05, ** *p* < 0.01.
