# Supplementary figures and images for: The Monocytes That Repopulate in Mice After Cyclophosphamide Treatment Acquire a Neutrophil Precursor Gene Signature and Immunosuppressive Activity
Source: Front Immunol. 2021 Jan 25;11:594540. doi: 10.3389/fimmu.2020.594540 (PMC7868404; doi:10.3389/fimmu.2020.594540)

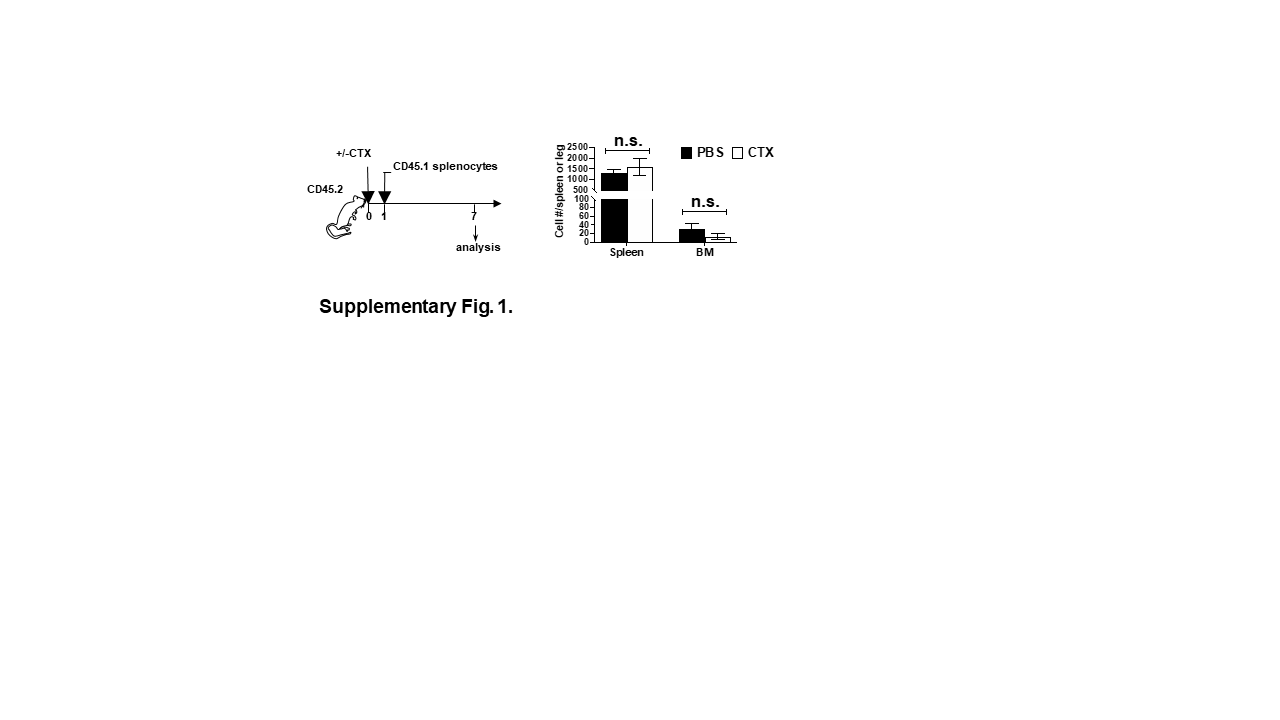

Supplement: Supplementary Figure 1 — Splenic HSCs do not undergo local expansion in the spleens of CTX-treated mice. Following the timeline depicted in the schema, naïve BALB/c (CD45.2+/+) mice were treated with PBS or CTX (150 mg/kg). The next day, a total of 50 million splenocytes derived from CD45.1+/+ mice were intravenously injected into each recipient. 7 days after PBS or CTX treatment, spleens and BMs were collected and processed for flow cytometry analysis. The numbers of Lin-cKit+Sca-1+ HSCs in donor cells (CD45.1+) shown as mean ± SD were summarized in bar graphs with three mice in each group. n.s. not significant. [file Image_1.tif]

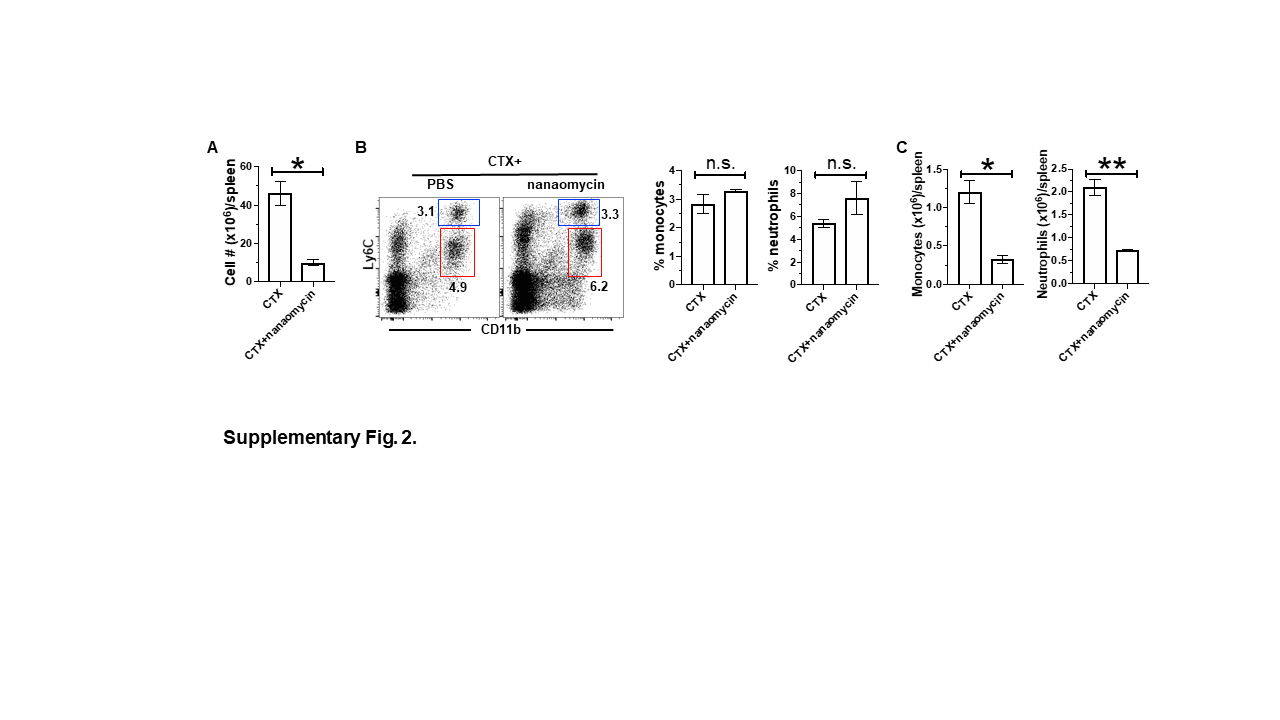

Supplement: Supplementary Figure 2 — DNMT3b inhibition does not impair CTX-driven myelopoiesis. Naïve BALB/c mice were treated with CTX. At indicated time points (day 1, 3, 5 after CTX), a cohort of mice were ip injected with nanaomycin A (200 µg/injection). 7 days after CTX treatment, spleen cells were harvested for analysis. (A) The total spleen cell numbers under each condition are summarized in bar graph with at least three mice per group. (B) Representative dot plots shown indicate the presence of myeloid cell subsets in spleen, and the numbers indicate the frequencies of gated populations. The frequencies of each myeloid subset are summarized in bar graphs with at least three mice each group. (C) The bar graphs summarize the numbers of monocytes and neutrophils under the indicated condition with at least three mice per group. n.s. not significant, * p < 0.05, ** p < 0.01. [file Image_2.tif]
